# Systems Analysis of Bioenergetics and Growth of the Extreme Halophile *Halobacterium salinarum*

**DOI:** 10.1371/journal.pcbi.1000332

**Published:** 2009-04-03

**Authors:** Orland Gonzalez, Susanne Gronau, Friedhelm Pfeiffer, Eduardo Mendoza, Ralf Zimmer, Dieter Oesterhelt

**Affiliations:** 1 Department of Membrane Biochemistry, Max-Planck Institute for Biochemistry, Martinsried, Germany; 2 Institute for Informatics, Ludwig-Maximillians-University Munich, Munich, Germany; 3 Physics Department and Center for Nanoscience, Ludwig-Maximillians-University Munich, Munich, Germany; 4 Computer Science Department, University of the Philippines, Diliman, Philippines; University of Duisburg-Essen, Germany

## Abstract

*Halobacterium salinarum* is a bioenergetically flexible, halophilic microorganism that can generate energy by respiration, photosynthesis, and the fermentation of arginine. In a previous study, using a genome-scale metabolic model, we have shown that the archaeon unexpectedly degrades essential amino acids under aerobic conditions, a behavior that can lead to the termination of growth earlier than necessary. Here, we further integratively investigate energy generation, nutrient utilization, and biomass production using an extended methodology that accounts for dynamically changing transport patterns, including those that arise from interactions among the supplied metabolites. Moreover, we widen the scope of our analysis to include phototrophic conditions to explore the interplay between different bioenergetic modes. Surprisingly, we found that cells also degrade essential amino acids even during phototropy, when energy should already be abundant. We also found that under both conditions considerable amounts of nutrients that were taken up were neither incorporated into the biomass nor used as respiratory substrates, implying the considerable production and accumulation of several metabolites in the medium. Some of these are likely the products of forms of overflow metabolism. In addition, our results also show that arginine fermentation, contrary to what is typically assumed, occurs simultaneously with respiration and photosynthesis and can contribute energy in levels that are comparable to the primary bioenergetic modes, if not more. These findings portray a picture that the organism takes an approach toward growth that favors the here and now, even at the cost of longer-term concerns. We believe that the seemingly “greedy” behavior exhibited actually consists of adaptations by the organism to its natural environments, where nutrients are not only irregularly available but may altogether be absent for extended periods that may span several years. Such a setting probably predisposed the cells to grow as much as possible when the conditions become favorable.

## Author Summary

Living cells can produce usable energy through various means. For example, animals derive energy, through respiration, from nutrients that they consume, and plants from light using photosynthesis. The particular microorganism that we study, *Halobacterium salinarum*, is a model organism for the archaeal domain of life. It is bioenergetically flexible in that it can perform both respiration and photosynthesis and in addition can also derive energy using fermentation. Accordingly, it is a good model system for investigating the interplay between different energy generating mechanisms. In this study, we investigate these relationships as well as how energy production is linked to the other processes involved in growth, including the consumption of nutrients and the production of cellular material. Because *Halobacterium salinarum* thrives in salt-saturated solutions, such as those that may be found in salt lakes and solar salterns, our study yields insight on how these cellular processes operate in environments that are lethal to most life on Earth.

## Introduction

*Halobacterium salinarum* is a halophilic archaeon that thrives in extremely saline environments with salt concentrations reaching 4 M or higher. The organism is perhaps most well known for its retinal-protein bacteriorhodopsin (BR), which is a light-driven proton pump. BR is the only known nonchlorophyll structure that allows photosynthesis [1]. It is currently being developed for applications in optical security [2], optical data storage [3], and holography [4]. Accordingly, *H. salinarum*'s photosynthetic capabilities are its most well-studied aspects. For example, the 3D structure of BR has been resolved, and its complete catalytic cycle elucidated at the molecular level (reviewed in [5]). However, despite the focus on BR, photosynthesis is not the only means by which *H. salinarum* can generate energy. Respiration [6],[7] as well as the fermentation of arginine [8],[9] are other mechanisms utilized by the organism. This bioenergetic flexibility makes the archaeon a good model system for investigating the interplay between different energy production modes. *H. salinarum* is also one of the few reported organisms that can use potassium gradients for long term energy storage in a battery-like manner [10].

The metabolic network of an organism can be reconstructed from genomic, biochemical, and physiological data [11],[12],[13]. This network consists of the known and hypothesized reactions that take place within the organism, and is considered to be on a genome-scale when most or all of the genes with known metabolic function are included [14]. We, in a previous study, have reconstructed and proposed such a network for *Halobacterium salinarum* [15]. In addition to the immediate information gained from metabolic reconstructions, these networks can be analyzed to gain insights on emergent system properties through the use of appropriate computational methods. In this respect, the constraints-based framework has emerged as an important and convenient tool for modeling such systems because it does not require the detailed information typically required by full kinetic models. Rather, constraints-based models require only generally available physicochemical information such as stoichiometry, reversibility, energy balance, and, when available, reaction velocities [16],[17],[18].

One of the methods available under the constraints-based framework is Flux Balance Analysis (FBA). Essentially, FBA uses linear optimization to find a flux distribution that maximizes a particular objective function, e.g., growth rate or ATP production [19],[20]. It has been shown that such optimality principles, within limits and under defined conditions, can describe the operation of metabolic networks, including the prediction of internal fluxes [21],[22]. Extensions to FBA include hybrid models that introduce some degree of dynamics through the integration of time-variant input rates to the static model [15],[19],[20].

Our aim in this study is two-fold. First, we set out to investigate the interplay between energy generation, nutrient utilization and biomass production under different bioenergetic modes. Second, we also analyzed the relationships between the different energy producing mechanisms of respiration, photosynthesis and fermentation themselves, which are typically examined individually. To achieve these, we used a genome-scale metabolic network that connects the different aspects. Our results include several findings that are contrary to assumptions which are typically made; particularly with respect to the utilization of nutrients, and how the bioenergetic modes operate. From a more methodological perspective, we also sought out to extend the existing framework for hybrid genome-scale metabolic models to handle biological systems where nutrient utilization and growth rates vary with time. Such changes in nutrient consumption, for example, can be the result of the differences between growth phases, or can arise from the interactions between the supplied metabolites. We demonstrate that the extended methodology not only accounts for such dynamics, but in several instances actually led to the identification of the underlying causes.

## Results/Discussion

### The Respiratory Chain

**Figure 1:**
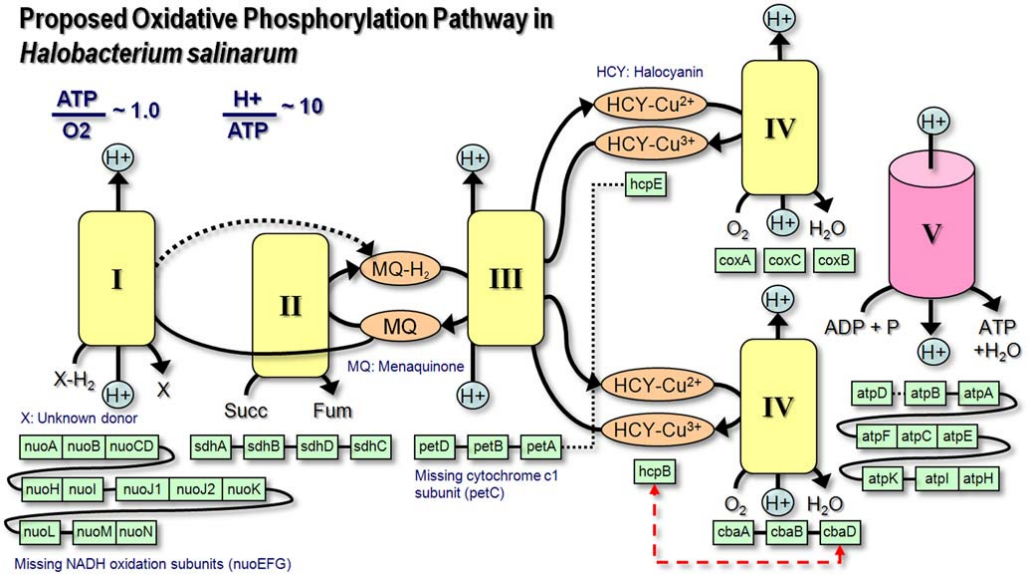
Proposed oxidative phosphorylation pathway. *H. salinarum* has analogs of all five respiratory complexes found in mitochondria and *E. coli* (complexes I to V). The boxes beneath each complex represent genes coding for specific subunits, which are often encoded adjacently in the genome (indicated by solid connections or by shared borders). Black broken lines are used to indicate that the connected genes, while not adjacent, are in the same genetic vicinity. The red broken line indicates that the *cbaD* and *hcpB* genes are fused in the archaeon. The proposed pathway has some notable differences from its more well-studied counterparts in *E. coli* or mitochondria. For example, genes coding for cytochrome c, which normally carries electrons to the terminal oxidase, could not be found in *H. salinarum*. Experimental evidence indicates that the function is likely performed by the copper protein halocyanin.

Most of the reactions in the genome-scale metabolic network we use in this study are taken directly from previous reconstructions [15],[23]. After updating some pathways, the final network is now composed of 664 reactions (567 internal and 97 transport)—covering 478 genes—and 545 metabolites. One of the most significant pathways that was modified is the respiratory chain. The proposed oxidative phosphorylation pathway is shown in Figure 1. *H. salinarum* has analogs of all five complexes associated with oxidative phosporylation found in mitochondria and *E. coli* (complexes I through V). However, there are significant differences from its better-studied counterparts. For example, the subunits of complex I that comprise the NADH acceptor module (nuoEFG) could not be assigned, similar to what has been observed for *Sulfolobus solfataricus* [24]. Indeed, it has been experimentally excluded that NADH is oxidized by a type I dehydrogenase in *H. salinarum*. Rather, NADH is oxidized by a non-homologous type II NADH dehydrogenase that also reduces quinones but is incapable of proton translocation [6]. Nevertheless, the conservation of eleven complex I subunits with high levels of sequence similarity make it likely that the complex is functional and translocates protons. Moreover, the lack of the NADH-specific acceptor module and the experimental evidences that NADH is not oxidized by complex I make it likely that the complex I analog actually accepts electrons from another donor molecule, which we left unspecified in Figure 1. In addition, the *H. salinarum* pathway is also different in that menaquinone, rather than ubiquinone, is the likely mobile carrier used to shuttle electrons from the complex I analog to the complex III analog. A similar proposal has been made for the closely related organism *Natronomonas pharaonis* [25],[26].

Two other important differences of the proposed pathway from the systems in mitochondria and *E. coli* are the composition of the complex III analog and the mobile carrier which carries electrons to the terminal oxidase (complex IV analog). The cytochrome *c1* subunit (*petC*) of *E. coli* complex III, responsible for transfering electrons to the mobile carrier of the organism, cytochrome c, does not seem to have a homolog in *H. salinarum*. In fact, genes which code for cytochrome c could also not be assigned. Accordingly, we believe that *H. salinarum* likely uses a different carrier. We propose this to be halocyanin, which is a blue copper protein originally isolated from *N. pharaonis* [27]. A similar function has been proposed for the molecule in *N. pharaonis*, based on its localization as a membrane protein and its midpoint potential that is consistent with a mobile carrier's [25]. This proposed function is further supported by the fact that the halocyanin gene, *hcpB*, is fused with the *cbaD* subunit of complex IV in *H. salinarum* [28].

Very little is known regarding the stoichiometry of the proton translocating processes in the respiratory chain of *H. salinarum*. Fortunately, while information on the individual components are unavailable, data on the aggregate process of respiration exists. This is very important for modeling because it determines the overall energy production capability of the organism. O_2_ pulse experiments indicated an ATP to O_2_ ratio of 1∶1. Measurements of initial proton uptake during phosphorylation demonstrated a ratio of 10∶1 between H^+^ and ATP [29]. These values are consistent with the experimentally determined photosynthetic stoichiometries of 22 photons per ATP and 2 photons per H^+^, given that light inhibits respiration with an observed stoichiometry of 24 photons per O_2_ molecule [7],[29]. We fixed the stoichiometry of the oxidative phosphorylation pathway in the model according to these values.

### Aerobic Growth

Consumption and production rates were modeled using differential equations of the form of either Equation (2) or Equation (3) (*cf.* Methods), with the rational that the production or consumption of a metabolite depends on the availability of the metabolite, the population size, and the current growth rate; note that growth-rate is time-varying in batch cultures. The piecewise extension of Equation (2), Equation (3), was necessary because several metabolites exhibited distinct modes over the growth period. For example, alanine, at some point, switched from production to consumption. We obtained parameters for each nutrient using both equation forms. To minimize the possibility of overfitting, we used the piecewise form in the final model only if it resulted in at least a reduction of 10% in residual error, which is a measure of the (dis-)agreement between model and data (see methods). In such a case, the boundary parameter 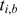 of the equation, which is optimized and automatically obtained, indicates a time near where the qualitative change in the uptake pattern occurs.

**Figure 2:**
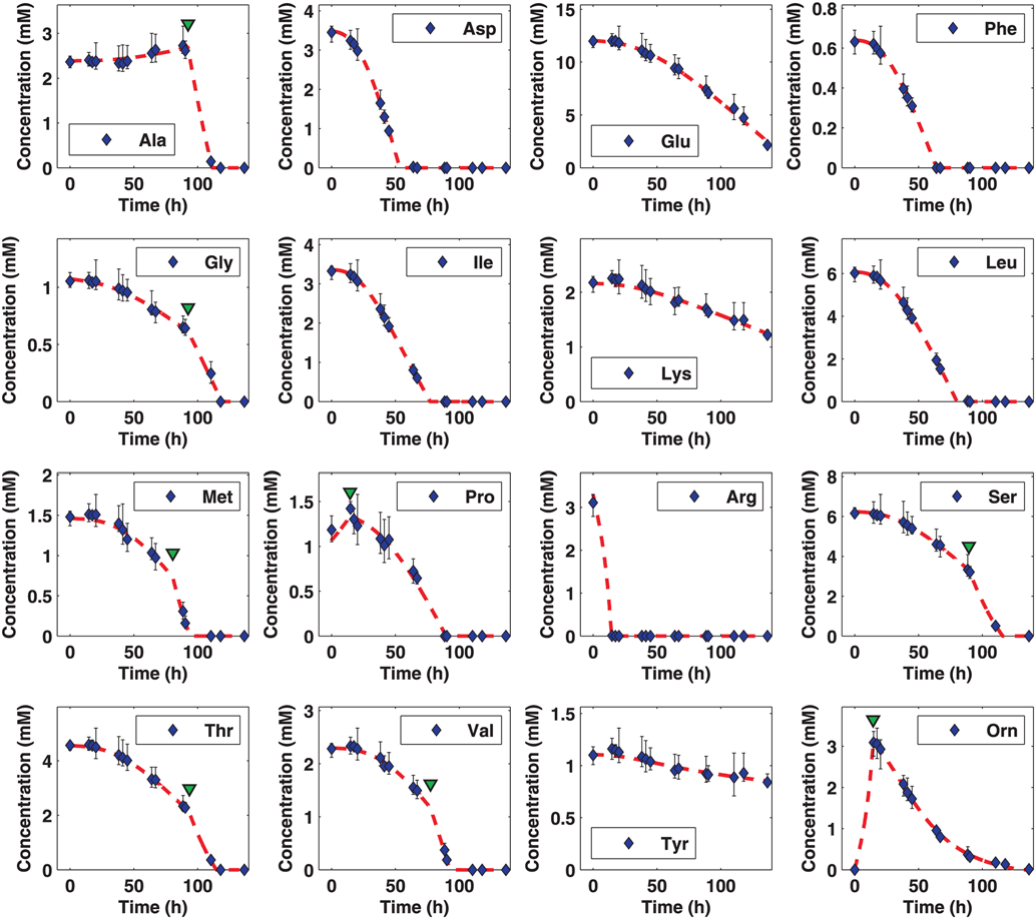
Nutrient consumption and production data from aerobically grown cells. Experimental data are shown using diamonds (average) with error bars provided. Model simulations are illustrated using red broken curves. The transport patterns of several metabolites qualitatively change during growth. For example, alanine and ornithine switch from production to consumption. For such metabolites, the piecewise Equation (3) was used to model utilization, and the corresponding 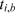 parameter value, which represents a point near where the qualitative change occurs, is indicated with an inverted green triangle.

Table 1 lists the best (lowest) residual error values we obtained using equations (2) and (3) for each nutrient (see methods). From the table, it is clear that the piecewise form hardly makes any difference for a number of metabolites, including aspartate, isoleucine, and leucine. For these three amino acids, residual error values only decreased by 0.8%, 0.2%, and 1%, respectively. Given that the same three amino acids, after arginine, are also the ones with the highest uptake rates, it would seem that they are the preferred or primary metabolites of *H. salinarum* during aerobic growth, at least among the nutrients supplied. Unlike most of the other provided metabolites, the uptake patterns of these amino acids remained constant, and their consumption rates high, up until their depletion. The experimental nutrient utilization data and the corresponding model simulations are shown in Figure 2.

**Table 1:** Comparison of transport equation forms.

Nutrient	Basic	Piecewise	Error reduction
Alanine	0.3946	0.0300	92.4%
Aspartate	0.0135	0.0133	0.8%
Glutamate	0.4211	0.3947	6.3%
Phenylalanine	0.0009	0.0009	1.0%
Glycine	0.0075	0.0043	43.2%
Isoleucine	0.0171	0.0171	0.2%
Lysine	0.0245	0.0227	7.3%
Leucine	0.0599	0.0593	1.0%
Methionine	0.0144	0.0112	22.4%
Proline	0.0367	0.0167	54.5%
Serine	0.1810	0.1299	28.2%
Threonine	0.0803	0.0575	28.4%
Valine	0.0196	0.0128	35.0%
Tyrosine	0.0087	0.0082	6.6%
Ornithine	2.3873	0.0325	98.6%

The transport rate of each compound was modeled using a differential equation of the form of either Equation (2) or Equation (3). The latter equation form, which is a piecewise version of the former, was necessary because the uptake patterns of several nutrients qualitatively change during growth. Columns 2 and 3 above list the best (lowest) residual error values for Equation (2) and Equation (3), respectively. The rightmost column indicates how much the error is reduced by using the piecewise version over the simpler form.

**Figure 3:**
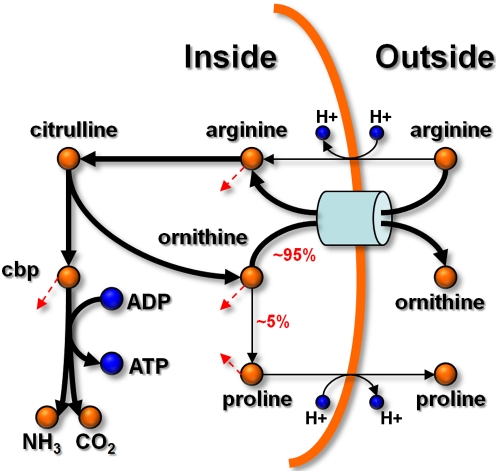
Arginine, proline and ornithine metabolism. Prior to its depletion, arginine exhibits the highest uptake rate among the supplied nutrients. Most of it is deaminated to citrulline, which is then converted to ornithine and carbamoyl-phosphate (cbp). Most of the ornithine (≈95%) is transported outside through an arginine-ornithine antiporter. On the other hand, carbamoyl phosphate is primarily degraded to NH_3_ and CO_2_ in a reaction that produces ATP from ADP. Simulations show that at the beginning of growth this process produces most of the energy in the cells. The red broken arrows indicate connections to other parts of the network.

In contrast to aspartate, leucine and isoleucine, the piecewise uptake equation form is clearly superior over the basic definition (Equation 2) for some metabolites. Most notable of these are ornithine, alanine and proline, for which residual errors dropped by 98.6%, 92.4% and 54.5%, respectively. Each of these amino acids, at some point, switches from production to consumption. For ornithine and proline, it is interesting that the points at which their respective utilization patterns change are close to each other, and that both occur near the time when arginine is depleted. This makes sense as arginine is consumed rapidly by the cells, and both ornithine and proline are downstream of its catabolic route. Flux-balance simulation at a point before arginine depletion shows the situation depicted in Figure 3. Most of the arginine, which at the beginning exhibits the highest uptake rate, is deaminated to citrulline via the action of *arginine deiminase* (EC 3.5.3.6; OE5208R). Citrulline is then converted to ornithine and carbamoyl-phosphate by *ornithine carbamoyltransferase* (EC 2.1.3.3; OE5205R). Ornithine is mainly transported outside through an arginine-ornithine antiporter (≈95%), a process that accounts for most of the arginine taken up. On the other hand, carbamoyl-phosphate, via the action of *carbamate kinase* (EC 2.7.2.2; OE5206R), is degraded to NH_3_ and CO_2_ in a reaction that produces ATP from ADP. In summary, most of the supplied arginine is rapidly converted to ornithine through a process that produces ATP. As will be discussed later, this fermentation process accounts for most of the energy in the cells at the early stages of growth.

The uptake rate of valine accelerates at 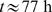, near the point where isoleucine is depleted. Similarly, the uptake rate of methionine accelerates at 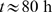, near the time when leucine is depleted. While it is hard to conclude with certainty, given the resolution and quality of the current data, that the acceleration of valine uptake actually precedes that of methionine, it is very likely that the increased consumption rates for both are the result of cells compensating for the depletion of leucine and isoleucine. Note that the two depleted branched-chain amino acids seem to be preferred metabolites of *Halobacterium salinarum* during aerobic growth. Accordingly, cells had to switch their metabolism to utilize more of the substitute sources in order to sustain growth. Alternative explanations are provided as supplementary information (Text S1).

The other supplied metabolites with utilization patterns that we found to exhibit distinct modes are alanine, serine, threonine and glycine. The first switches from gradual production to rapid consumption, and the latter three demonstrate uptake rates that significantly accelerate. In each of these cases, the critical point seems to be near 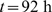, which is also near the time when proline, methionine and valine are depleted. Although we believe that the increased consumption rates are also compensatory measures for the depleted metabolites, it is again difficult to conclude this with certainty, given the current data. Moreover, if true, it is also difficult to determine whether which nutrients serves to balance for what. Considering the significant qualitative interpretations that the 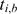 parameters carry, we performed further steps aimed at analyzing the quality of the obtained values. We provide these results as supplementary information (Text S1).

**Figure 4:**
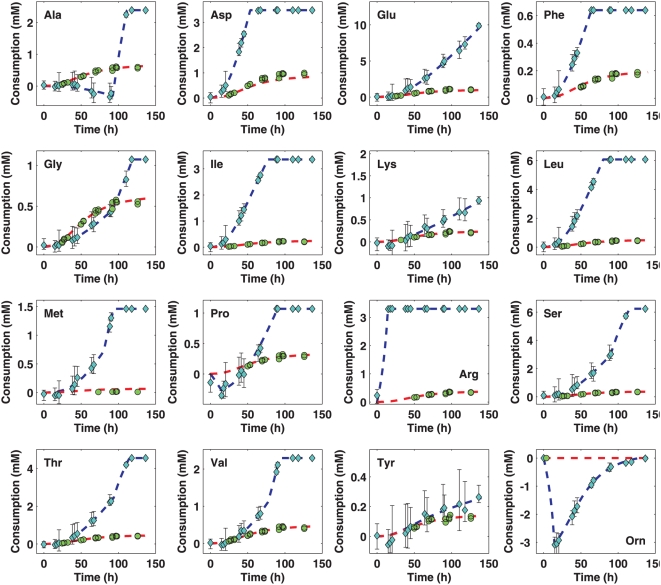
Summary of nutrient uptake and incorporation rates under aerobic conditions. The blue curves indicate the net amount of each amino acid that has been consumed (positive) or produced (negative) as a function of time. The red curves, on the other hand, show the total amount of each that has been incoporated into the biomass, whether integrated into proteins or as free metabolites. Clearly, for most of the supplied amino acids, the rate at which they are taken up from the medium far exceeds the rate at which they are directly incorporated. This implies that the pertinent amino acids are, in addition, significantly catabolized for energy, and/or are used to synthesize the other biomass constituents that are not supplied.

Nutrients consumed by cells can have various fates: (1) they can be incorporated into the biomass with at most very minimal changes, such as in the case of amino acids incorporated as protein residues or free metabolites; (2) they can be converted to other biomass components, typically after partial degradation; (3) they can be oxidatively degraded to CO_2_ for the production of energy through respiration; and (4) they can be secreted after conversion to another metabolite, for example following partial degradation. Given that the rates at which most of the supplied amino acids were consumed far exceeded the requirements for biomass incorporation as described in the first fate mentioned above, then at least one of the other three possible fates must also be true for the relevant amino acids. The results for individual metabolites are summarized in Figure 4, and the comparison between total (global) carbon uptake and total carbon incorporation (fates 1 and 2) is provided as supplementary information (Figure S1).

Consistent with our previous observations [15], leucine, isoleucine, valine and methionine are among the nutrients which are mostly degraded, whether for energy or as carbon skeleton donor. This is remarkable because the four are, along with lysine, essential amino acids for *H. salinarum*. The heavy rates at which they are degraded can cause the organism to prematurely terminate its own growth. One would typically assume that it would be better for the cells to use essential nutrients sparingly, especially considering the fact that there are other non-essential sources of energy and carbon available. However, leucine and isoleucine, as discussed earlier, are even among the preferred metabolites of *H. salinarum*, i.e., nutrients for which the highest uptake rates were observed.

All nutrients that are taken up by cells are either incorporated into the biomass (fates 1 and 2 mentioned above) or converted to metabolic by-products (fates 3 and 4). By approximating the biomass composition using measurements, calculations and assumptions, and then correlating these information with the observed population sizes, analysis and prediction of the latter set of fates becomes possible. We took most of the biomass composition values from our previous work [15], which includes the contribution of individual amino acids, nucleotides, lipids, sugars, and several other metabolites. However, we repeated the determination of the amino acid content, which accounts for over 60% of the total organic mass, using measurements at various optical densities. The results are summarized in supplementary Figure S2. While the relationships between the amino acids and the optical density are not strictly linear, average errors from linear fits are below 12.5% in all cases. Most of the deviations are situated in the lower optical density range. With respect to the previous set of measurements [15], the most significant difference of the current one is that the total amino acid content is about 30% higher at approximately 503 µg/OD·ml. Possible factors that could have contributed to this discrepancy include differences in the conditions used, including media composition, and the fact we more rigorously adapted the current cultures. The discrepancy is well within the range of inaccuracy that may be introduced by morphological changes in the cells to the relationship between biomass and optical density. Accordingly, we also adjusted the biomass requirement (composition) of the other constituents.

CO_2_ is an expected metabolic by-product of cells under aerobic conditions. Indeed, if it is assumed that all carbon atoms that are taken up and do not appear in the biomass are completely degraded through respiration, then CO_2_ should account for the bulk of the by-product pool. We calculated the (theoretical) amount of oxygen that will be needed under such a scenario at various points during growth, using flux balance analysis where we set energy production as the objective function and assumed an unlimited oxygen supply. Note that stoichiometric relationships between the oxidation of carbon substrates and the consumption of oxygen are implicitly defined by the reactions (pathways) in the metabolic network. However, comparison of these calculated results with actual oxygen consumption measurements indicated that respiratory-linked degradation is not the fate of most carbon atoms that are consumed but do not get incorporated into the biomass. In fact, the data shows that cells use only about 20% of the oxygen that they would otherwise need to completely oxidize all the material. Thus, the formation and accumulation of other by-products in significant quantities (in the mM range), probably including those that result from forms of overflow metabolism, likely occur in the preparations. In addition, the described situation already seems to have been the case even before the oxygen supply became limiting at 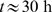. The computations are summarized in Figure S3 (supplemental information). Further flux analyses show that the most efficient by-products with respect to energy production are common intermediates such as acetate and succinate.

We observed a maximal respiratory rate of approximately 1 µmol O_2_/OD·ml·hr during growth. At about 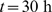, the amount of dissolved oxygen in the medium was reduced to 0%. Given that prior to this point, large amounts of nutrients that are consumed but neither incorporated into the biomass nor subjected to oxidative phosphorylation could already be observed, the respiratory process itself is likely the bottleneck prior to 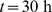. Past this point however, cells could only respire in at most the rate at which oxygen dissolved into the medium. Note that a 0% oxygen saturation level does not mean that oxygen is no longer available to the cells because flasks were kept open. Given that growth continued well past the point when the oxygen supply started to become limiting, the respiratory rate dropped steadily from then on.

Under aerobic conditions, cells can derive energy (ATP) through respiration and substrate level phosphorylation. We calculated the maximum (theoretical) energy that the system can produce as well as the fraction of this that can be attributed to respiration at various points, using the observed nutrient utilization. The values are reflected by the red and blue curves, respectively, in Figure S4 (supplemental information). The system maximum energy was calculated as
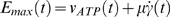
(1)where 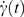 is the current growth rate, and 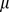 is the growth-related energy that is implicitly taken into account by the metabolic network in synthesizing the compounds included in the biomass [30]. In order to find flux distributions that maximize energy production, we introduced an ATP hydrolysis reaction (ATP+H_2_O = ADP+P) into the network, and performed flux balance analysis with it defined as the objective function. 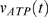 in Equation (1) refers to the flux through this reaction.

Throughout growth, the theoretical maximum energy that the system can produce (red curve in Figure S4) is significantly higher than the energy produced by respiration (blue curve). Although it is possible that the actual energy production of the system is closer to the respiratory curve, it is clear that prior to the depletion of arginine (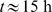), the earlier described fermentation process for this amino acid accounts for most of the energy produced by the system. Evidently, the energy generated by this process can be comparable to the two “primary” modes (respiration and photosynthesis), contrary to what is typically assumed. This is consistent with the fact that *H. salinarum* can be grown with neither oxygen nor light just as readily, by supplying large amounts of arginine. After the amino acid is depleted, respiration accounts for most of the produced energy. The additional energy that can be produced through the further non-respiratory-related degradation of other (non-arginine) nutrients is significantly smaller. This being the case, it is remarkable that non-respiratory-related processes account for approximately 80% of the nutrients that are consumed but not incorporated into the biomass. Again, we should note that the pertinent metabolites include essential ones.

**Figure 5:**
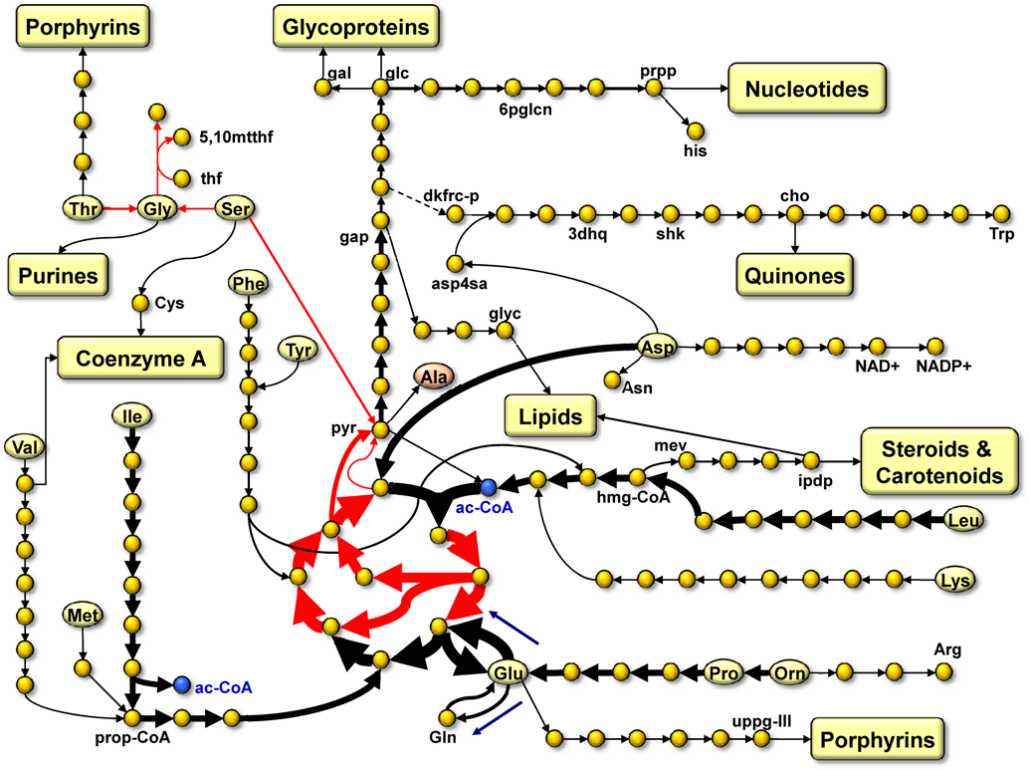
Predicted fluxome during exponential phase (*t* = *30*) that maximizes energy production. Yellow ellipses represent compounds that are taken up from the medium, while light-red ellipses represent those that are accumulated in the medium. Larger fluxes are drawn with thicker arrows. Black and red arrows correspond to qualitatively invariable and qualitatively variable (see main text) fluxes, respectively. Side reactants, for the most part, are not depicted in the figure. Yellow rounded boxes represent biochemical pathways. Metabolite abbreviations: 3dhq - 3-dehydroquinate, 5,10mtthf - 5,10-methylenetetrahydrofolate, 6pglcn - 6-phosphogluconate, ac-CoA - acetyl-CoA, Ala - alanine, Asn - asparagine, Asp - aspartate, asp4sa - aspartate 4-semialdehyde, Arg - arginine, Cys - cysteine, dkfrcp - 6-deoxy-5-ketofructose 1-phosphate, gal - galactose, gap - glyceraldehyde 3-phosphate, cho - chorismate, glc - glucose, Gly - glycine, glyc - glycerol, Glu - glutamate, Gln - glutamine, His - histidine, hmg-CoA - 3-hydroxy-3-methyl-glutaryl-CoA, ipdp - isopenteny diphosphate, Ile - isolecuine, Leu - leucine, Lys - lysine, Met - methionine, mev - mevalonate, Orn - ornithine, Phe - phenylalanine, Pro - proline, prop-CoA - propanoyl-CoA, prpp - 5-phosphoribosyl diphosphate, pyr - pyruvate, Ser - serine, shk - shikimate, thf - tetrahydrofolate, Thr - threonine, Trp - tryptophan, Tyr - tyrosine, uppg-III - uroporphyrinogen III, Val - valine.

We computed fluxes that are consistent with the observed consumption and production rates during the exponential phase (specifically 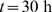) and which additionally maximize energy production. These are illustrated in Figure 5. In it, compounds which are taken up from the medium are represented by yellow ellipses, and compounds which are accumulated in the medium by red ellipses. Fluxes through reactions are presented using arrows, where thickness is used to indicate strength. Considering that it is uncertain to what extent energy production is actually the objective of cells *in vivo*, we used flux variability analysis [31] to complement the results by computing the minimum and maximum possible fluxes through each reaction after removing all optimality assumptions. Fluxes through reactions for which the values are either both positive or both negative are drawn in black. Intuitively, these are reactions that are constrained enough by the network stucture, pseudo-stability and the observed exchange fluxes, to have non-zero activity regardless of any optimality assumptions. That is, the network structure and the measured data are already enough to guarantee the existence of the fluxes and their indicated directions.

During the exponential phase 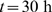), we found 378 (67%) of the 567 internal reactions of the metabolic network to have qualitatively invariable fluxes. Of these, 246 carry non-zero fluxes, and 132 are blocked under the conditions. The fact that measurements of a relatively few input and output fluxes already qualitatively determines the fluxes through most of the reactions is related to the bow tie structure of the metabolic network [15],[32]. Catabolic pathways, which together form one fan of the bow tie, fan-in into the knot of central metabolism to supply the common intermediates. These pathways are highly linear and convergent in structure. Accordingly, nutrients which are taken up and not directly incorporated into the biomass will have to go through the associated catabolic pathway with no variability until the level of the common intermediates is reached. Similarly, biosynthetic pathways, which form the other fan of the bow tie, fan-out and branch from the knot of central metabolism, and are also highly linear in structure. Thus, most biosynthetic routes of cellular constituents will also be invariable once past the level of the metabolic core.

### Phototrophic Growth

Phototrophic cultures were grown under conditions similar to the aerobic preparations, except that oxygen was absent and they were illuminated with white light. We were able to observe final optical densities well above 1.5 OD, comparable to the population levels achieved by their counterparts (see Figure S5). The most apparent difference is with respect to having slower growth rates. While aerobic preparations exhibited exponential phase doubling times of only roughly 11 hours, phototrophic cultures demonstrated times close to 50 hours. The reason for this disparity is unclear. Representative aerobic and phototrophic growth curves are provided as supplementary information (Figure S5). In both cases, the preparation depicted is the result of three rounds of adaptation by iterative reinoculation into the respective condition.

We modeled the consumption and production of nutrients during phototrophy using the same procedure we employed for the aerobic case. Accordingly, the 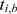 parameter, when applicable, again reflects the point at which qualitative change in the uptake pattern of the respective nutrient occurs. The experimental data and the corresponding model simulations are provided as supplementary information (Figure S6). Analogous to the aerobic case, arginine was rapidly depleted near the start of growth, again with most of it being converted to ornithine and secreted by cells into the medium. This is interesting because arginine fermentation genes (*arcABC*) have been found to be repressed during phototrophy, presumably to repress the secondary energy source [33]. However, the rapid depletion of arginine and the equally rapid accumulation of ornithine is a clear indication that the fermentation process was in fact active despite the phototrophic condition of the cells. Considering that we used inoculants taken from cultures already growing under phototrophy, it is unlikely that the observations are only due to the time (lag) that is required for regulation to take effect.

Next to arginine, aspartate exhibited the highest uptake rate, and is the second supplied amino acid that was depleted at 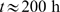. Up to that point, very little, if at all, glutamate uptake could be observed. However, consumption of the amino acid either starts or accelerates appreciably after the exogenous aspartate supply is exhausted. Similarly, methionine uptake also seems to accelerate at this point. Some 200 hours later (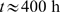), phenylalanine is depleted, and threonine and serine are nearly exhausted. Also at about the same time, alanine switches from production to consumption, and the uptake of glycine accelerates. The three amino acids glycine, serine and threonine are connected to each other through their degradative pathways. Presumably, when glycine is no longer produced through the degradation of excess amounts of either serine or threonine, then cells have to take up more of it. This is consistent with the observation that prior to this point, glycine uptake is significantly lower than the rate at which the amino acid is incorporated into the biomass whether as protein residues or free metabolites (Figure S7), not even considering the fact that glycine, via tetrahydrofolate, is also used as methyl donor in various biosynthetic pathways.

Unlike in the aerobic case, the depletion of phenylalanine coincided with a number of discernable qualitative changes in the uptake patterns of the nutrients. In addition to the ones stated above, consumption of the amino acid serine seems to have stopped at about the same time, even though growth, albeit already in the late exponential phase, could still be observed. The reason for this is unclear. Serine was already nearly exhausted at about 0.2 mM by then. Moreover, it is also at this point that alanine switched from production to consumption. Serine and alanine are metabolically related to each other through their degradative pathways, as the two can enter central metabolism through pyruvate. In the aerobic case, not only were we unable to observe an arrest in serine uptake, but the switch of alanine from production to consumption actually conincided with the acceleration of serine consumption. While neither serine nor alanine share the catabolic route of phenylalanine, it is possible that phenylalanine played a more important role in the phototrophic case; that is, the observed behaviors may have been indirect effects of the amino acid's depletion. In this respect, we should note that the early biosynthetic steps of aromatic amino acids are shared with the quinones, which play critical roles in respiration.

Analogous to the aerobic case and somewhat unexpectedly, the rates at which most of the supplied amino acids were taken up from the medium far exceeded the rates at which they were incorporated into the biomass with minimal modifications (fate 1). These findings are summarized in Figure S7. Again, the implication is that the consumed quantities of the pertinent amino acids are considerably catabolized by the cells. Certainly, significant portions of these are used as building blocks (carbon skeleton donors) for the production of other biomass constituents (fate 2), such as nucleotides and lipid molecules. However, even the substrate requirements for the synthesis of these unsupplied biomass components are not enough to explain the differences. This can readily be seen in Figure S8, which shows the total carbon uptake against the total carbon incorporation under the conditions. Accordingly, similar to the aerobic case, other metabolites (by-products) are also likely produced and accumulated in considerable quantities. This is somewhat more unexpected during phototrophy than in respiration because the necessary oxidative breakdown of nutrients in the latter is not present. Under phototrophic conditions, one would typically expect material consumption to be only near the quantity sufficient for biomass production because energy can already be derived from light, rather than from the supplied metabolites. The energy yield of the non-respiratory degradation of these materials should be very small compared to the energy produced through photosynthesis, especially considering that arginine is the only fermentative substrate of *H. salinarum*. Nevertheless, in retrospect, this behavior is consistent with the emerging picture that the organism takes an approach toward growth that is focused on the here and now, even at the cost of longer-term concerns. Indeed, the essential amino acids leucine, lysine, isoleucine, methionine and valine are again among the amino acids that are taken up in amounts far more than required for biomass formation.

When phototrophic cultures reached 1.5 OD, only about 14% of the supplied carbon that had been consumed could be attributed to the amino acid content of the biomass (directly measured; see Figure S8). This means that the total carbon incoporation rate was only about 20% (accounting for the other biomass components: eg., nucleotides, lipids, etc…). In comparison, when aerobic cultures reached comparable levels of population (also in the late exponential phase), we could attribute about 13% of the consumed carbon to the amino acid content of the biomass (directly measured; see Figure S1), which implies a total carbon incorporation rate of approximately 19%. This means that about 80% and 81% of the consumed carbon were not incorporated in the phototrophic and aerobic cases, respectively. Correcting the aerobic value to account for the respiratory-related oxidative degradation of nutrients—using a 1∶1 CO_2_ to O_2_ ratio which was calculated using the stoichiometry of the parts of the metabolic network that are involved in the oxidation of the relevant amino acids—shows that approximately 66% of the carbon was neither incorporated nor respired. Considering the fact that the growth rates observed for phototrophy were nearly five-fold slower, it is remarkable that the ratios of the nonincorporated, nonrespired carbon under both conditions are as close to each other as they are. Indeed, aerobic cultures already reached 1.5 OD after only slightly more than 80 hours, while, in stark contrast, phototrophic cultures took about 450 hours to reach similar levels. It would therefore seem that a large part of the nutrients that were taken up but were neither incorporated into the biomass nor used as respiratory substrates has more to do with growth (biomass production) than with any form of maintenance. For this reason, it is likely that some as of yet uncharacterized, non-maintenance growth processes are at least partially responsible for the low carbon incorporation rates.

### Environmental Adaptations

Several unexpected findings were made during the course of this study. These include: (1) that essential amino acids are degraded not only during respiration but even under phototrophic (anaerobic) conditions, where energy should already be abundant; (2) that fermentation of arginine, which is often considered a secondary, alternative energy source, occurs simultaneously with either respiration or photosynthesis; (3) that considerable amounts of metabolites are produced and accumulate in the medium under both conditions, as a result of nutrients that are consumed but not incorporated into the biomass nor used as respiratory substrates; and (4) in connection with the previous point, that the total carbon incorporation rate is extremely low even under phototrophic (anaerobic) conditions (approximately 20%), when one would typically expect nutrient consumption to be in quantities that are only sufficient for biomass production since energy is already derived from light. All of these findings are consistent with the seemingly “greedy” behavior demonstrated by *Halobacterium salinarum* that we noted in our previous study [15], which we believe actually consists of adaptations to its natural environment, where nutrient availability is not only irregular but can also be absent for extended periods of time.

In the salt lakes and solar salterns where *Halobacterium salinarum* may be found, life is characterized by blooms that may not occur for years after a previous episode. In the Dead Sea for example, no growth of the unicellular green alga, *Dunaliella parva*, which is responsible for all of the primary productivity, is possible when the salt concentration of the water column is invariably high. Blooms of *Dunaliella* in the Dead Sea occur only after significant dilution of the upper levels by the influx of freshwater. In turn, this allows the archaeal community to bloom at the expense of the organic material produced by the alga [34]. The fact that the conditions under which these blooms are possible may not be realized for years after a previous episode [35],[36] may have inclined *Halobacterium salinarum* to grow as much as possible when the conditions become favorable. In connection to this, as for the times when their environments are not conducive to growth, the capacity of the halobacteria to survive in adverse conditions for extended periods has been well established [37],[38]. This capacity allows them to survive until the next bloom. In this respect, we should note that a considerable number of the reported viable ancient cells that have been recovered are at least moderately halophilic (halotolerant) [39],[40],[41].

### Future Work

One of the predictions of our model is that non-CO_2_ by-products/metabolites accumulate in the medium in significant amounts under both aerobic and photorophic conditions, likely including partially degraded forms of the supplied nutrients. Unfortunately, attempts to identify the secreted molecules are complicated by the (necessary) extremely high salt concentrations (4 M) in the growth media. We intend to focus on the identification of these metabolites and on the elucidation of the reasons/dynamics behind their accumulation in a succeeding study.

Specific sets of nutrients dominate material uptake at various points during growth. For example, at the early stages, highest uptake rates were observed for aspartate and leucine, and, to a lesser extent, glutamate and serine. Note that arginine uptake was the highest, but most of its carbon skeleton was secreted as ornithine. With this information in hand, we would like to perform flux analysis using labeled substrates [22] in order to obtain quantitative constraints for at least a fraction of the internal fluxes. This should allow for a more detailed view of the metabolic strategy used by the cells.

## Materials and Methods

### Hybrid Flux Balance Model

For the consumption or production of each nutrient 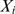, we used a simple model consisting of three terms:
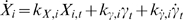
(2)where 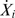 is the uptake rate of nutrient 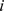, 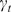 is the population size at time 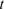, 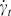 is the current growth rate defined as 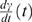, and 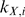, 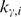 and 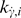 are optimizable parameters. The rational of the construction is that the production or consumption of a metabolite depends on the availability of the metabolite, the population size and the current growth rate.

Preliminary inspection of the data revealed that the transport patterns of some of the metabolites change during growth. For example, ornithine, at some point, switches from being produced and accumulated in the medium to being steadily consumed by cells. Accordingly, we used a simple extension of Equation (2) that allows for two modes:
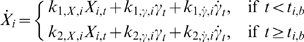
(3)where 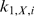, 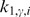, 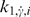, 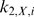, 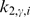, 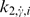 and 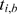 are optimizable parameters. Similar to the basic form, the consumption or production of each metabolite still depends on the availability of the metabolite, the population size and the current growth rate. However, the new parameter 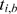 now separates time into two intervals, and the distinct set of parameters for each of these allows the utilization pattern to qualitatively change in moving from the first to the second.

For each nutrient 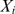, parameters for both Equations (2) and (3) were estimated from experimental data using the *fminsearch* function of MATLAB. Systematically defined sets of initial parameter values were used in solving the inverse problems (parameter estimation). To minimize the possibility of overfitting, the more sophisticated form of Equation (3) was used in the final model, in favor over the simpler equation form, only if it resulted in significant improvement, that is, the residual error, a measure of the (dis-)agreement between the model and data, is substantially lower. In this respect, it is reasuring that although Equation (3) is clearly better for some metabolites, such as alanine and ornithine, it hardly makes any improvement for others, such as leucine and isoleucine, which seem to be staples of the cells (see results). In cases where Equation (3) is used, the optimized parameter 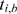, intuitively, corresponds to a point near where the uptake pattern changes qualitatively. Note that the borders (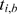) need not be equal for all *X_i_*'s. Because the choice between the two equation forms can be done automatically, the computational system we employ not only accounts for changes in metabolite modes, but can actually lead to the detection and identification of these modes, and, subsequently, to the recognition of the biological processes behind them.

A metabolic network can be conveniently represented as a stoichiometric matrix **S**, where each row corresponds to a metabolite and each column to a reaction. The entries of **S** are the stoichiometric coefficients that define the relationships between the reactions and compounds. A positive value for 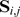 indicates that compound 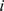 is produced in the left to right direction of reaction 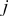, while a negative value indicates that it is consumed. For a particular time interval during growth with length 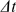, a set of fluxes which are consistent with the observed nutrient depletion/accumulation and biomass formation can be obtained by solving the linear program
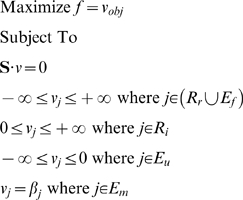
(4)where 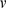 is a vector of fluxes defining the flux 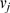 through each reaction 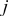, 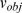 is the objective function, 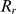 is the set of reversible internal reactions, 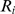 is the set of irreversible internal reactions, 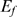 is the set of exchange fluxes associated with ubiquitous metabolites, and 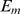 is the set of exchange fluxes that correspond to experimentally measured nutrients. The set of ubiquitous compounds include CO_2_, H_2_O, Na^+^, Cl^−^ and H^+^. For each reaction 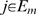, the value 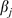 is the appropriate evaluation of the corresponding uptake equation (2 or 3) for the interval. In this work, all of the supplied carbon and energy sources, except for citrate, were included in 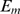. In addition, the set also includes ornithine, which is initially accumulated in the medium, and oxygen. Biomass production is treated in a similar manner by fixing the flux of the growth (pseudo-)reaction to observed rates. To account for the possibility that cells produce and accumulate in the medium certain metabolites, for example in the case of overflow metabolism, the set of one-way exchange reactions, 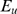, was included. It includes central metabolites such as acetate, pyruvate, malate and GAP, nucleotides, and some sugars such as glucose. Unless otherwise specified, energy production was used as the objective function.

### Experimental Protocols

Strain *Halobacterium salinarum* R1 (DSM 671) cells were grown in chemically-defined medium, with composition defined in Table S1. The medium composition that we used is the simplest known for the halophile that allows population levels comparable to those reached when using complex media. Preparatory cultures were grown in 100 ml flasks containing 35 ml of the medium to a cell density of ≈1 OD, from which 1 ml inoculants were taken to start the next culture. This was done repeatedly to adapt cells to their growth conditions. All cultures were prepared in flasks which had side arms to measure turbidity (cell density) via a Klett photometer, and were carried out in duplicates. Cell suspensions were shaken at 105 rpm at 40°C in the dark. At specific points, samples were taken from the cultures so that 14–18 samples were collected over the growth period, and these were stored at 4°C. To separate the cells from the medium, the samples were centrifuged for five minutes at 15,000 rpm, using a SS34 rotor. Pellets were resuspended in 500 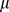
l basal salt (medium without the amino acids) and spun down as before. Amino acid analysis was performed on both the pellets and the original supernatants, using an Amino Acid Analyzer (Biotronik LC3000). Oxygen saturation in the medium was monitored using the “Fibox 3-trace v3, fiber-optic oxygen meter” from Precision Sensing GmbH (Regensburg, Germany). Calculation of the actual oxygen consumption rates is provided as supplementary information (Text S2). Similar protocols were used for phototrophically grown (anaerobic) cells, except that flasks were first flushed with nitrogen to remove oxygen, and were closed using air-tight septa. To maintain the anaerobic condition of cultures while taking samples, syringes with a long needle, inserted through the septa, were used.

## Supporting Information

Figure S1 Total carbon consumption and biomass incorporation of aerobically grown cells. The green circles indicate the amount of carbon in the biomass that is due to amino acids (primarily protein). To account for the fact that amino acids (proteins) are not the only components of the biomass, we approximated the total carbon incorporation (inclusive of the amino acids) by scaling (multiplying) the biomass reaction definition, which is described in the section titled “Aerobic Growth” and characterized in (Gonzalez *et al*., 2008), with the population size. This total incorporation value is represented by the red broken curve. To allow for a comparison between material uptake and incorporation, we use the blue curve to indicate the total amount of carbon that has disappeared from the medium, calculated using the supplied nutrients and ornithine (filled triangles are experimental data). At *t* = 140 h, only about 19% of the total carbon that has been consumed can be accounted for by the biomass. A similar graph for the phototrophic case is provided in Figure S8.
(0.02 MB EPS)

Figure S2 Amino acid composition of biomass during aerobic growth. The amount of each individual amino acid in the biomass was quantified at different optical densities. The measurements include those that are integrated into proteins (residues) and those that are free inside the cells. The red line in each graph indicates the best linear fit for the particular molecule. Average deviation from the line in all cases is below 12.5%. Due to experimental limitations, we were not able to obtain values for cysteine and tryptophan, and the values for aspartate and glutamate are already inclusive of asparagine and glutamine, respectively.
(0.04 MB EPS)

Figure S3 Theoretical (energy-optimal) and actual oxygen consumption rates. The red broken curve indicates the amount of oxygen that would have been needed if all nutrients that were taken up but did not get incorporated into the biomass were used for respiration. The blue broken curve shows the actual oxygen utilization rate. The large discrepancy between the two curves suggest that overflow metabolism is prevalent. The oxygen supply starts to become limiting at about *t* = 30 h. The smaller peaks and troughs of the red curve are due to the discontinuities of the piecewise models used for the uptake of several metabolites.
(0.01 MB EPS)

Figure S4 System energy in ATP equivalent. The red curve shows the maximum amount of energy, computed using FBA, that the system can produce at various points during growth. The blue curve indicates the amount of energy that is produced through respiration. Interestingly, prior to the depletion of arginine (*t* {similar, tilde operator } 15), fermentation of the amino acid generates most of the energy in the system, even more than respiration.
(0.01 MB EPS)

Figure S5 Growth curves. (Left) Cells grown aerobically in the dark. A doubling time of approximately 11 hours was observed. (Right) Cells grown anaerobically in light. The culture exhibited a much slower growth rate, with a doubling time of about 50 hours. Both cultures are the result of three rounds of adaptation by repeated inoculation to the respective condition.
(0.02 MB EPS)

Figure S6 Nutrient consumption and production data from cells grown anaerobically in light. Experimental data are indicated using diamonds (average) with error bars provided. Model simulations are illustrated using red broken curves. The transport patterns of several metabolites qualitatively change during growth. For such metabolites, the piecewise Equation (3) was used to model utilization, and the corresponding *ti*,*b* parameter value, which represents a point near where the qualitative change occurs, is indicated with an inverted green triangle. This figure is similar to Figure 2, except that that was for the aerobic condition.
(0.15 MB EPS)

Figure S7 Summary of nutrient uptake and incorporation rates under phototrophic (anaerobic) conditions. The blue curves indicate the total amount of each amino acid that has been consumed/produced. The red curves show the total amount of that particular amino acid that has been incoporated into the biomass, whether integrated into proteins or as free metabolites, as calculated from the population size and the growth (biomass) function. Similar to the aerobic case, and somewhat unexpectedly, most of the supplied amino acids exhibited uptake rates that far exceeded the rates at which they were incorporated. This implies that the amino acids, in addition to biomass incorporation, are also catabolized significantly.
(0.24 MB EPS)

Figure S8 Total carbon consumption and biomass incorporation of phototrophically grown cells. The green circles indicate the amount of carbon in the biomass that is due to amino acids (primarily protein). To account for the fact that amino acids (proteins) are not the only components of the biomass, we approximated the total carbon incorporation (inclusive of the amino acids) by scaling (multiplying) the biomass reaction definition, which is described in the section titled “Aerobic Growth” and characterized in (Gonzalez *et al*., 2008), with the population size. This total incorporation value is represented by the red broken curve. To allow for a comparison between material uptake and incorporation, we use the blue curve to indicate the total amount of carbon that has disappeared from the medium, calculated using the supplied nutrients and ornithine (filled triangles are experimental data). At *t* = 650 h, only {similar, tilde operator }20% of the total carbon that has been consumed can be found in the biomass.
(0.02 MB EPS)

Table S1 Composition of chemically-defined medium.
(0.02 MB PDF)

Text S1 Analysis of boundary parameters.
(0.11 MB PDF)

Text S2 Oxygen measurements.
(0.05 MB PDF)
