# Supplementary material for: Systems Analysis of Bioenergetics and Growth of the Extreme Halophile Halobacterium salinarum
Source: PLoS Comput Biol. 2009 Apr 3;5(4):e1000332. doi: 10.1371/journal.pcbi.1000332 (PMC2674319; doi:10.1371/journal.pcbi.1000332)
Supplement: Table S1 — Composition of chemically-defined medium. (0.02 MB PDF) [file pcbi.1000332.s009.pdf]

Table S1: Composition of chemically-defined medium

Description	Value	Description	Value
NaCl	4 M	L-Ala	2.5 mM
KNO_3	1 mM	L-Arg	3.5 mM
KCl	27 mM	L-Ile	3.4 mM
$MgSO_4$ * $7H_2O$	81 mM	L-Met	1.3 mM
Sodium Citrate * H_2O	1.7 mM	L-Pro	0.9 mM
K_2HPO_4	0.42 mM	L-Phe	0.8 mM
KH_2PO_4	0.58 mM	L-Ser	5.8 mM
$FeSO_4$ * $7H_2O$	11.6 μM	L-Thr	4.2 mM
$CuSO_4$ * $5H_2O$	0.2 μM	L-Tyr	1.1 mM
$MnCl_2$ * $4H_2O$	1.8 μM	L-Val	2.5 mM
$ZnSO_4$ * $7H_2O$	1.5 μM	L-Lys	2.1 mM
Na_2MoO_4 * $2H_2O$	0.1 μM	Thiamin	16.5 μM
L-Asp	3 mM	Folate	11.5 μM
L-Glu	13.5 mM	Biotin	2.1 μM
L-Leu	6.1 mM	pH	7.2
L-Gly	1 mM		
