# Supplementary material for: Systems Analysis of Bioenergetics and Growth of the Extreme Halophile Halobacterium salinarum
Source: PLoS Comput Biol. 2009 Apr 3;5(4):e1000332. doi: 10.1371/journal.pcbi.1000332 (PMC2674319; doi:10.1371/journal.pcbi.1000332)
Supplement: Text S1 — Analysis of boundary parameters. (0.11 MB PDF) [file pcbi.1000332.s010.pdf]

S1 Analysis of $t_{i,b}$ parameters

The consumption and/or production of a metabolite with a utilization pattern that qualitatively changes during growth was modeled using Equation (2). The $t_{i,b}$ parameter of this equation carries significant qualitative interpretation because it indicates a point near where the qualitative change occurs. Accordingly, we performed further steps to check how well-defined the values we obtained are, given the available data. Specifically, for each appropriate metabolite X_i, we fixed $t_{i,b}$ to specific values, and optimized for the best residual error that can be achieved leaving all the other parameters free. As with before, we used systematically defined initial guesses (for the other parameters). The results are summarized in Figure S9. In the figure, we plot for each metabolite the best residual error value as a function of $t_{i,b}$ (red broken curves). The values inidicated are normalized to the overall minimum, and therefore good values for $t_{i,b}$ are points at which the curve is near 1.0 (low).

Through manual inspection we found that most of the $t_{i,b}$ parameter values fall at points that are near the time of depletion of at least one other supplied metabolite. For example, the $t_{i,b}$ values initially obtained for ornithine and proline, where both metabolites switch from production to consumption, are near the point at which arginine is depleted. Similarly, the $t_{i,b}$ value of valine is near the depletion point of isoleucine. This can readily be seen by comparing Figures 2 and S9. In such cases, we manually synchronized the events by adjusting the $t_{i,b}$'s. The final values we used are indicated by the blue broken lines in Figure S9.

It is clear that a global $t_{i,b}$ parameter value is not possible because of metabolites like ornithine that plainly switch mode before the rest. Nevertheless, arguments relating to model simplicity may be made, given the current data, for using a common $t_{i,b}$ value for all metabolites that change modes later in the growth period, particularly alanine, glycine, methionine, serine, threonine and valine. Such synchronization could, for example, be the result of a global (metabolic) shift that occurred at that time, which induced the observed mode changes in the metabolites. While such an interpretation remains a possibility, it is inferior to the current interpretation that most of the mode changes are simply compensatory measures for the depletion of other nutrients in a number of ways. The reasons include: (1) From Figure S9, it can be seen that there is no single value for which the consumption/production models of the relevant nutrients are all at their best, i.e., each is at a point where the residual error is lowest. (2) Also from Figure S9, it is difficult to reconcile the idea that the qualitative changes occur nearly simultaneously with the observation that the curves of the relevant metabolites are quite diverse in their shapes; for example, some curves reach their lowest point (1.0) a lot earlier than others, and also begin rising earlier than others. If the events were indeed synchronized, then one would expect the curves to be more similar than they are. And (3) the best synchronized parameter value, if ever one is to be used, is around $t = 87.5$. In this vicinity, only two of the relevant curves are at a region where the residual error is significantly higher than the corresponding lowest value. The window is actually quite narrow, such that moving even by a couple of hours in either direction would mean that at least one metabolite is already at a point where its residual error is more than 50% worse than its best. However, the problem with using a value within this vicinity to synchronize the mode changes, in addition to the ones mentioned above, is that it would mean that the total material uptake of cells from the time when major metabolites, such as leucine and isoleucine, are depleted up until the critical point will be relatively very low compared to the rest of the growth period. Moreover, given that all of the relevant mode changes are described by either an acceleration in uptake rate or a switch from production to consumption, then the total material uptake starting from the critical point on will be exceedingly high. Indeed, for methionine and valine, an acceleration that takes place as late as $t \approx 87.5$ would imply an incredibly high uptake rate because both should already be depleted by about $t = 92$. We should note that an abrupt increase in total material uptake is not something that is to be expected of a culture that is slowing down with respect to growth (late log phase). Finally, nutrient substitution has been described in bacteria and archaea. For example, *E. coli* has been shown to start consumption of acetate once the glucose supply is depleted (Varma and Palsson, 1994; Mahadevan *et al.*, 2002).

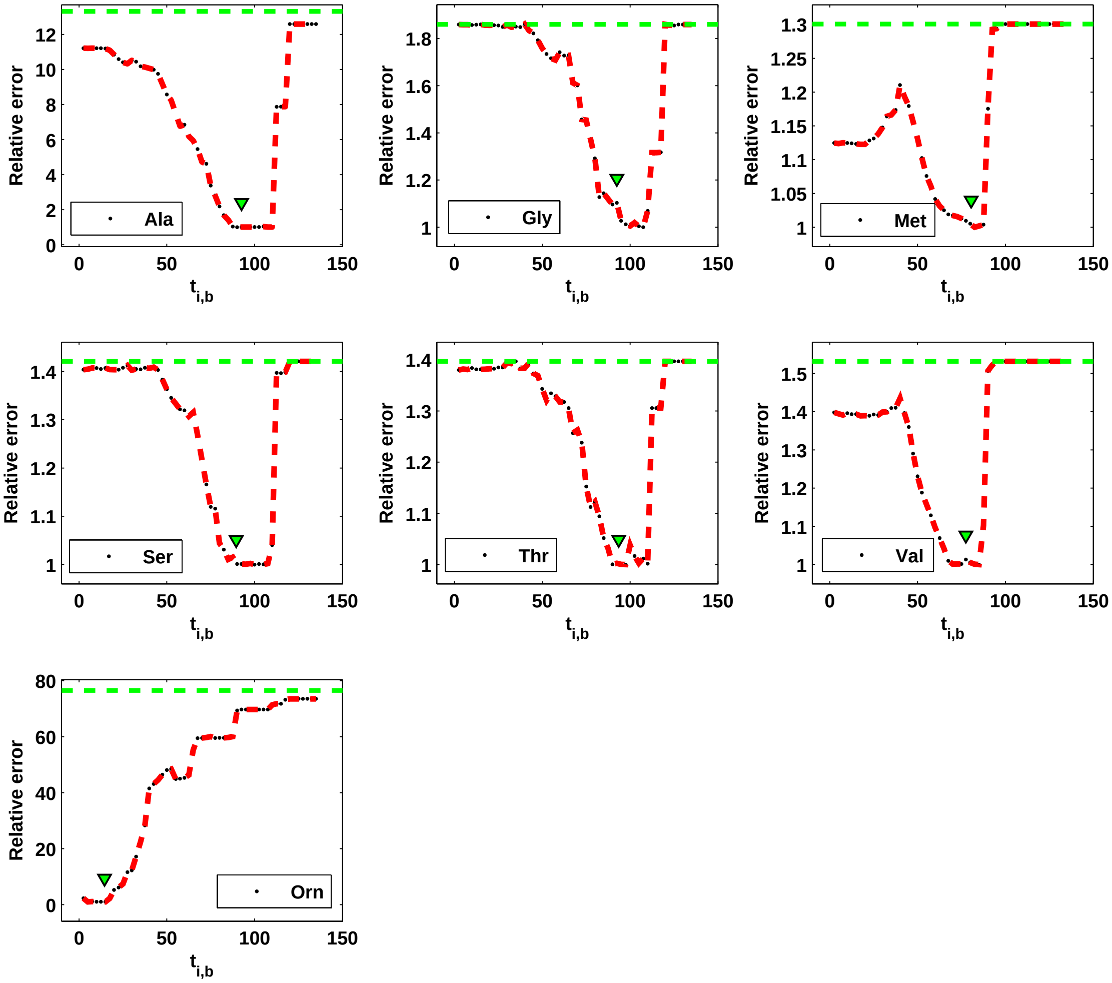

Figure S9: Parameter ($t_{i,b}$) exploration for aerobically grown cells. The transport patterns of several of the supplied metabolites qualitatively change during growth. For such a nutrient, the point at which the change occurs is represented by the $t_{i,b}$ parameter of Equation 2. The above graphs show for each metabolite the best residual error we were able to obtain for specific values of $t_{i,b}$ normalized to the overall minimum (red broken curve). As such, ideal values for $t_{i,b}$ should be points where the curve is near 1.0 (lowest point). The actual parameter value we used in the final model is indicated with an inverted, green triangle. For comparison, we also indicate with a green, horizontal line the best residual error value we were able to obtain using the non-piecewise equation form.

References

Mahadevan, R., Edwards, J., and Doyle, F. (2002). Dynamic Flux Balance Analysis of Diauxic Growth in *E. coli*. *Biophys. J.*, **83**, 1331–1340.

Varma, A. and Palsson, B. (1994). Stoichiometric flux balance models quantitatively predict growth and metabolic by-product secretion in wild-type *Escherichia coli* W3110. *Appl. Environ. Microbiol.*, **60**, 3724–3731.
