# Supplementary material for: Systems Analysis of Bioenergetics and Growth of the Extreme Halophile Halobacterium salinarum
Source: PLoS Comput Biol. 2009 Apr 3;5(4):e1000332. doi: 10.1371/journal.pcbi.1000332 (PMC2674319; doi:10.1371/journal.pcbi.1000332)
Supplement: Text S2 — Oxygen measurements. (0.05 MB PDF) [file pcbi.1000332.s011.pdf]

S2 Oxygen measurements

The amount of dissolved oxygen in the medium (solution) was continuously monitored using the "Fibox 3-trace v3, fiber-optic oxygen meter" from Precision Sensing GmbH (Regensburg, Germany). Under conditions identical to aerobic cultures, argon was blown into a flask that contains only medium, until oxygen saturation dropped to zero. At that point, we removed the argon source and equilibrated the flask with air. We then used the rate at which oxygen dissolved into the medium to characterize oxygen dissolution kinetics. Specifically, the oxygen transfer rate (OTR) was defined as

$$OTR = \frac{dx}{dt} = k\left(x_{max} - x\right) \tag{1}$$

where x is the amount of dissolved oxygen, and x_{max} is the maximum value of x, which indicates saturation. The data and the model fit are shown in Figure S10. With the appropriate parameters for Equation (1), we then calculated the oxygen consumption rate of a culture at time t using

$$consumption(t) = k\left(x_{max} - x\right) + m \tag{2}$$

where m is the current rate at which x is changing in the culture. For the sake of completeness, we note that Equation (1) has the analytical solution

$$x = x_{max} - \left(x_{max} - x_0\right)e^{-kt} \tag{3}$$

where $x(0) = x_0$ is the initial condition.

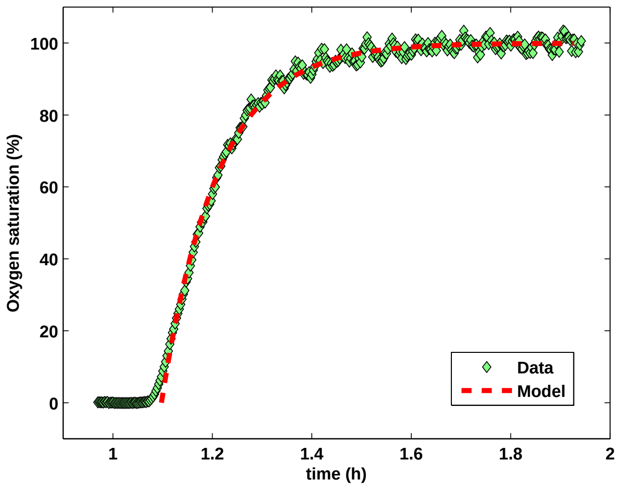

Figure S10: Dissolution kinetics of oxygen. The graph shows the velocity at which oxygen dissolves into the growth medium (cell-free) under the conditions used.
